# Influence of the Hierarchy Structure of Aluminum Particles on Density, Combustion Efficiency, and Ignition Delay

**DOI:** 10.3390/ma17174354

**Published:** 2024-09-03

**Authors:** Yaru Li, Liu Yin, Hui Ren, Xinzhou Wu, Jinshan Sun, Xuwang Liu

**Affiliations:** 1 State Key Laboratory of Precision Blasting, Jianghan University, Wuhan 430056, China; 2 Sichuan Hongbo Science and Technology Co., Ltd., Mianyang 621000, China; 3 State Key Laboratory of Explosion Science and Safety Protection, Beijing Institute of Technology, Beijing 100081, China; 4 Institute of Chemical Materials, China Academy of Engineering Physics, Mianyang 621900, China

**Keywords:** aluminum nanoparticles, core-shell structure, combustion efficiency, density

## Abstract

Aluminum nanoparticles (nAl) have received sustained interest due to their higher reactivity than micron aluminum particles (mAl). However, in practice, the densities of explosive formulations with nAl are far smaller than those with mAl, which greatly undercuts the energy release performance. To take advantages of both kinds of Al particles, in situ integration of mAl@nAl composites was proposed and evaluated. The mAl@nAl composites were prepared by in situ electrical explosion of Al wire. Their morphology, density, and specific surface area (SSA) were characterized by scanning electron microscope (SEM), densimetry, and Brunauer–Emmett–Teller (BET), respectively. SEM showed that nAl uniformly adhered to the surface of mAl. With the increase in voltage, the average diameter and density of the composites decreased, but the SSA of the composites increased. And the largest density of the composites was 1.13 g/cm^3^, comparable to that of the commercial graded Al product (1.25 g/cm^3^). Meanwhile, the highest SSA of the composites was 12.1192 m^2^/g. In addition, the combustion efficiency of mAl@nAl composites at 20 kV was 8.26% higher than that of physically graded counterparts. The constant-volume combustion test under zero oxygen balance revealed that the pressurization rate and peak pressure of mAl@nAl composites prepared at 20 kV were the highest of all. Furthermore, constant-volume combustion under constant heat showed that the combustion temperatures of mAl@nAl composites were 1.15–1.45 times higher than those of physically graded counterparts. Finally, the ignition delay of mAl@nAl composites was reduced with the increase in explosion voltage.

## 1. Introduction

Aluminum (Al) particles are some of the most economical and attractive fuel materials utilized in energetic materials fields, such as thermites, MEMS energy source, propellants, and explosive additives, to improve the energy content [1,2,3,4]. However, to date, the high theoretical energy content of Al particles has not been fully exploited, as the particle state (shape, grain size, and surface condition) significantly influences the reaction efficiency [5,6,7]. Various strategies have been used to modify the particle state to improve the reaction performance of Al particles. Streletskii et al. [8] activated Al through a mechanochemical strategy with graphite, and the reactivity of the resulting Al particles significantly exceeded that of standard aluminum particles. With the development of nanotechnology, nanometric Al (nAl) particles have received considerable attention in the combustion community. Researchers found that the reactivity of Al particles can be significantly enhanced by reducing the grain size of Al particles to the nanometric scale [9]. For example, the combustion behavior of nAl particles was quite favorable as compared with that of their micron-sized counterparts [10,11]. By adding nAl particles, the detonation velocity of explosives becomes much more favorable [9,12].

The loading density of explosive composites can significantly influence their detonation performances [13,14]. As Al particles have a larger density (2.7 g/cm^3^) than any kind of explosive compound (1.6–2.0 g/cm^3^), the density level of explosive composites can be greatly improved by adding Al particles. However, due to the low bulk density of nAl particles, the loading density of explosive composites with nAl particles is less desirable than that of explosive composites with their micron counterparts. Therefore, it is still a big challenge to take advantage of the high reactivity of nAl particles, and meanwhile keep the loading density of aluminized explosives as large as possible. To date, numerous efforts have been used to improve the performance of nAl particles, such as surface coatings with organics [15,16], polymers [17,18] and metals [5,19], and assembly with explosives [20,21]. These efforts may protect the reactivity of nAl particles, but little help is provided for improving the loading density.

To address this challenge, researchers explored the feasibility of mixing micro-sized aluminum (mAl) particles with nAl particles in traditional energetic formulations [22,23,24,25,26,27,28,29]. For example, Woody et al. [22] studied the reaction performance of the mixture of nAl and mAl particles by thermal analysis, and they found nAl particles and mAl particles influenced each other, and a direct reaction like sintering may occur between them. Moore et al. [23] replaced part of the mAl particles with 80 nm Al particles, and they found that, with increasing proportion of nAl particles, the combustion velocity of the thermite would be increased, whereas only with certain content of nAl particles could the ignition delay time of the thermite could be reduced. Huang et al. [28] investigated the flame propagation behavior of bimodal nano/micron sized aluminum particles with air, and they found aluminum particles with a small percentage of nAl showed a separated and wider flame regime. Jiang et al. [29] justified that the incorporation of nAl particles into mAl particles would enhance the reaction energy output of Al during the initial reaction stage of the explosion and increase the reaction ratio. Although those studies show that mixtures of nAl and mAl manifest their advantages, the preparation strategies lead to issues that cannot be neglected. On the one hand, the bimodal Al particles they used are of independent size distribution ranges, which will decrease the density level of the Al particles. On the other hand, the contact between nAl and mAl particles is not close enough to weaken the effect of nano-size during the reaction process. They both will cause great reductions in the reaction efficiency of the Al particles.

In the case of particulate application, one effective strategy to increase loading density is the particle gradation method. In theory, the more continuous the grain size distribution, the higher density of the composites that could be acquired [30]. Besides, continuous size distribution can also maximize the reaction efficiency of the Al particles. Under such circumstances, sustained energy release from the nAl reaction could be achieved from the initial stage of detonation to the far expansion field. Therefore, we managed to produce Al particles with continuous size distribution via in situ electrical explosion of Al wire. The density of the resulting particles was comparable to that of pure mAl particles. In addition, the reaction efficiency of Al composites was greatly improved as well.

## 2. Experimental

### 2.1. Mechanism of In Situ Preparation of mAl@nAl Composites

**Figure 1:**
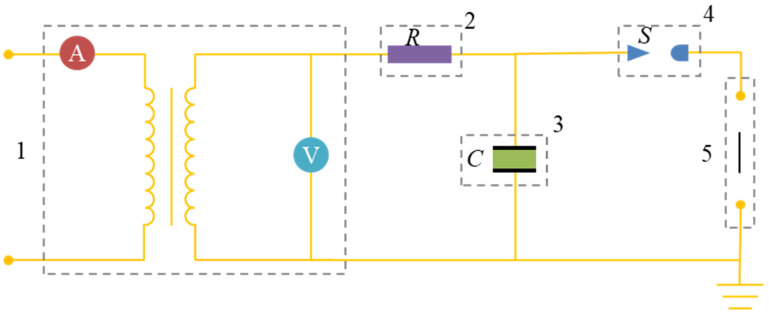
The electric circuit of a typical EEW set. 1 is the high voltage power supply; 2 is the resistance of the circuit; 3 is the capacitance; 4 is the switch; 5 is the discharge electrode.

**Figure 2:**
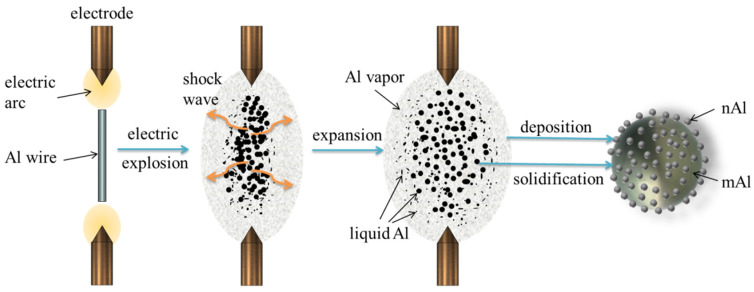
Diagram of mechanism of in-situ preparation of mAl@nAl composites.

Electrical explosion of wire (EEW) is a versatile approach to produce various of nanoparticles [31,32,33]. The typical circuit used for EEW is shown in Figure 1. The input energy is stored in the capacitor in advance. Once closing a switch, *S*, as shown in Figure 1, the electric energy will be transmitted to the wire in nanoseconds. For such a short time, the energy transfer process can be treated as adiabatic. Therefore, the energy received by the wire equals the stored energy, which can be calculated by Equation (1):

*E_in_* = 1/2*CU*^2^ (1)

where *C* is the capacitance, F; and *U* is the charging voltage of the capacitor. The received energy is larger than the enthalpy of vaporization (*E_v_*) of Al wire, which is calculated by Equation (2):

*E_v_* = *w_v_m*Δ*H_v_* (2)

where *w* is the mass percent of the vaporized Al wire, %; *m* is the mass of the Al wire, kg; and Δ*H_v_* is the vaporization enthalpy of per unit mass Al wire. The Al wire will experience melting, vaporization processes, and eventually condense to Al nanoparticles. However, the situation will be interesting if the input energy is not enough to vaporize the entire wire. Under such conditions, the skin layer of the wire would be heated first to the vapor state due to the skin effect of the current. Subsequently, the inner part is heated by the rest of the energy to liquefy, and then the liquid Al is scattered as droplets by the shock wave of the vapor, as shown in Figure 2. Eventually, mAl@nAl composites can be fabricated when gaseous Al is condensed on the Al droplets.

The energy distributed to the liquid Al is expressed by Equation (3):

*E*_l_ = *w*_l_*m*Δ*H_l_* (3)

where *w_l_* is the mass percent of the liquefied Al wire, %; *w_l_* + *w_v_* = 1; and Δ*H_l_* is the enthalpy of melting of per unit mass Al wire. Both the input energy and the Al wire parameters would influence the Joule heat distribution in the wire, which will further impact the size ratio of the particles. Therefore, it is significant to match the input energy with the Al wire parameters to acquire mAl@nAl composites with continuous size distribution.

### 2.2. Preparation of the mAl@nAl Composites

For the experimental set up, the capacitance was kept as 4 μF. Therefore, the input energy could be determined by the voltage of the capacitance. In this work, four different voltages and three kinds of aluminum wires (diameters: 0.55, 0.80, and 1.0 mm; Shijiazhuang Zhongli Zinc Industry Company, Shijiazhuang, China) were used to prepare the mAl@nAl composites to study the influences of the wire diameter and input energy on the properties of resulting composites. The resulting mAl@nAl composite was named after the voltage supplied to the wire. For example, if the supplied voltage was 14 kV, the corresponding mAl@nAl composite was named EX-14.

To make a comparison, aluminum composite with a comparable density level was also prepared by physical gradation of mAl particles and nAl particles. The size distributions of the particles were determined by referring to the industrial formulas, into which Al flakes were incorporated to guarantee the densities of the composites were comparable to those of the mAl@nAl composites. Accordingly, all physical graded composites were denoted as PG-content of nAl. For example, if the content of nAl in the composite was 20, it was denoted as PG-20. The detailed formulations are listed in Table 1.

### 2.3. Morphological and Compositional Characterization

The morphologies of the samples were characterized by a scanning electron microscope (SEM; S-4800, Hitachi, Santa Clara, CA, USA). The size distributions of the samples were analyzed by a laser particle size analyzer (LS 13 320, Beckman Coulter, Munich, Germany). The BET (Brunauer–Emmett–Teller) specific surface areas (SSAs) of the samples were acquired by a surface area analyzer (ASAP 2406, MicroActive, Norcross, GA, USA). The densities of the samples were characterized by a tap densimeter (BT-311, Better, Dandong, China). The active aluminum contents of the samples were determined by the gas volumetric method.

### 2.4. Combustion Performances

**Combustion heat under zero oxygen balance.** To evaluate the energy release performance of the samples, the combustion heats of the samples were tested under zero oxygen balance. The combustion heats of the samples were obtained by oxygen bomb calorimeter (TRHW-7000C, Tianrun, Weihai, China), with 0.500 ± 0.005 g sample ignited for each test.

**Figure 3:**
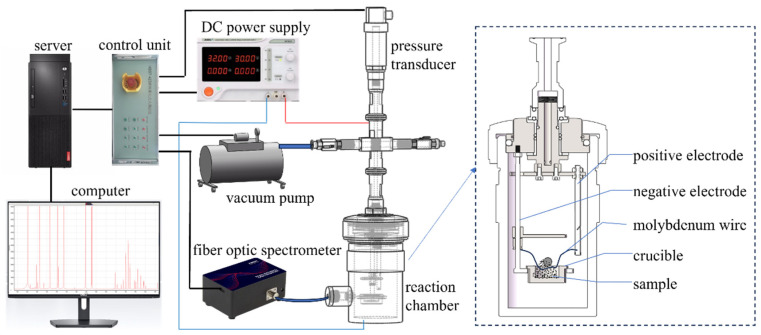
Schematic diagram of constant-volume combustion tests under zero oxygen balance.

**Constant-volume combustion tests under zero oxygen balance.** To examine the effect of hierarchy structure on the combustion efficiency, combustion tests under zero oxygen balance were performed. The tests were conducted in a homemade combustion bomb with a volume of 330 mL. Samples were ignited by 0.3 mm molybdenum wire under a supplied voltage of 60 V. In this study, ammonium perchlorate (AP) served as the oxidizer, and the weight ratio of AP and mAl@nAl composites was determined based on zero oxygen balance. Approximately 0.8 g sample was packed in the crucible for each test and was ignited under vacuum environment. The corresponding spectra of combustion products were recorded by fiber optic spectrometer with a measurable wavelength range of 200–1100 nm at a sampling frequency of 300 Hz. The schematic diagram of the test system is displayed in Figure 3.

**Constant-volume combustion tests at constant heat.** To characterize the influence of gaseous products on the pressurization action, constant-volume combustion tests at constant heat were carried out. For each test, the combustion heat was kept to 5 kJ, and hence the weight of the tested sample was determined based on the combustion heat. Meanwhile, the oxygen pressure was kept at 3 MPa. Due to the fast reaction rate, the heat loss in the process could be neglected. Under such conditions, the pressurization differences of the samples would be attributed to the temperature elevation.

**Figure 4:**
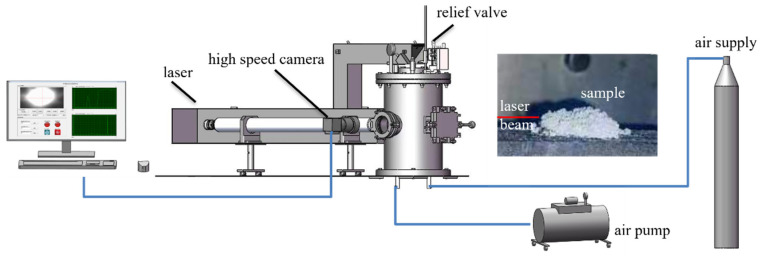
The schematic diagram of the laser ignition system.

**Ignition delay measurements.** The measurements were carried out in a 22 L closed bomb under air atmosphere. A CO_2_ laser was used to ignite the samples with a power of 60 W. A high-speed camera (CP70-1HS, Optronics International, Tulsa, OK, USA) was employed to record the ignition process. The sample was filled into a crucible. The ignition delay time was determined by the interval between the initiation of laser and a bright light emitted from the sample. A schematic diagram of the laser ignition system is presented in Figure 4.

## 3. Results and Discussion

### 3.1. Morphologies of mAl@nAl Composites

**Figure 5:**
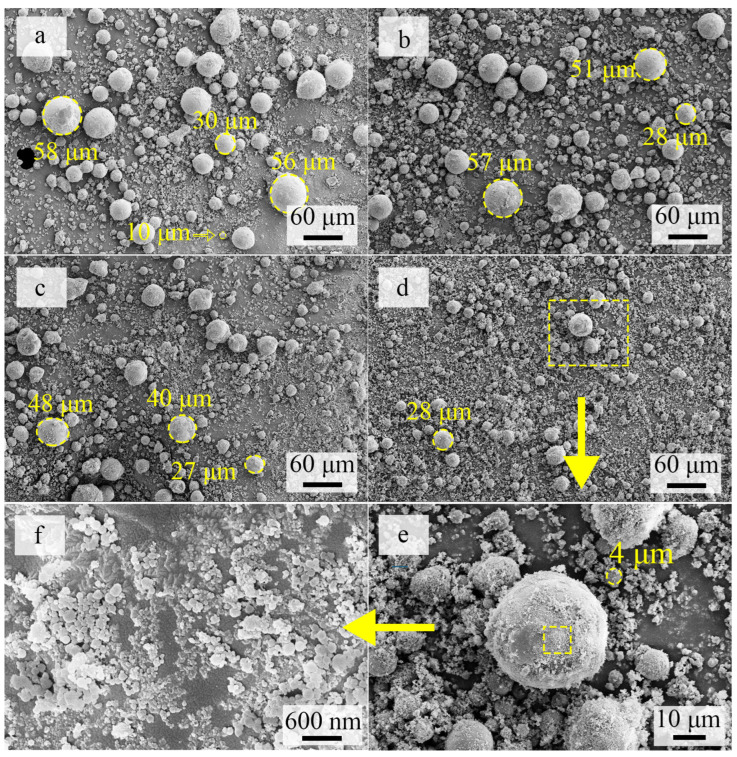
SEM images of mAl@nAl composites prepared under supplied voltages of (**a**) 14 kV, (**b**) 16 kV, (**c**) 18 kV, (**d**) 20 kV; (**e**,**f**) are the high-magnification SEM images of mAl@nAl composites prepared under a voltage of 20 kV.

Al wires with diameters of 0.55 mm, 0.80 mm, and 1.0 mm were separately tested under input voltages ranging from 14 kV to 20 kV. It was found that Al wires with diameters larger than 0.80 mm simply melted under the input voltage as large as 20 kV, and only millimeter-scale droplets were formed. Only in the case of Al wire of 0.55 mm could electric explosion be achieved, and the vaporization part increased with the increase in input voltage. SEM images of the resulting particles under different input voltages are shown in Figure 5.

As shown in Figure 5, the mAl@nAl particles prepared by in situ electric explosion were all spherical. The diameter of the particles ranged from around 60 μm to below 1 μm, as shown in Figure 5a, and particles over 10 μm took the majority. The average diameter of the resulting composite particles decreased with the increase in supplied voltage from 14 kV to 20 kV. Besides, the diameter of the largest particles was also gradually reduced in the same sequence. Ultimately, particles smaller than 10 μm took the majority, as shown in Figure 5d. Despite the size decrease, continuous size distributions of particles were achieved under all supplied voltages, as shown in Figure 5a–d. It could be seen from the high-magnification SEM images in Figure 5e,f that the surface of the micro-sized particles was uniformly covered by nano-sized aluminum particles.

**Figure 6:**
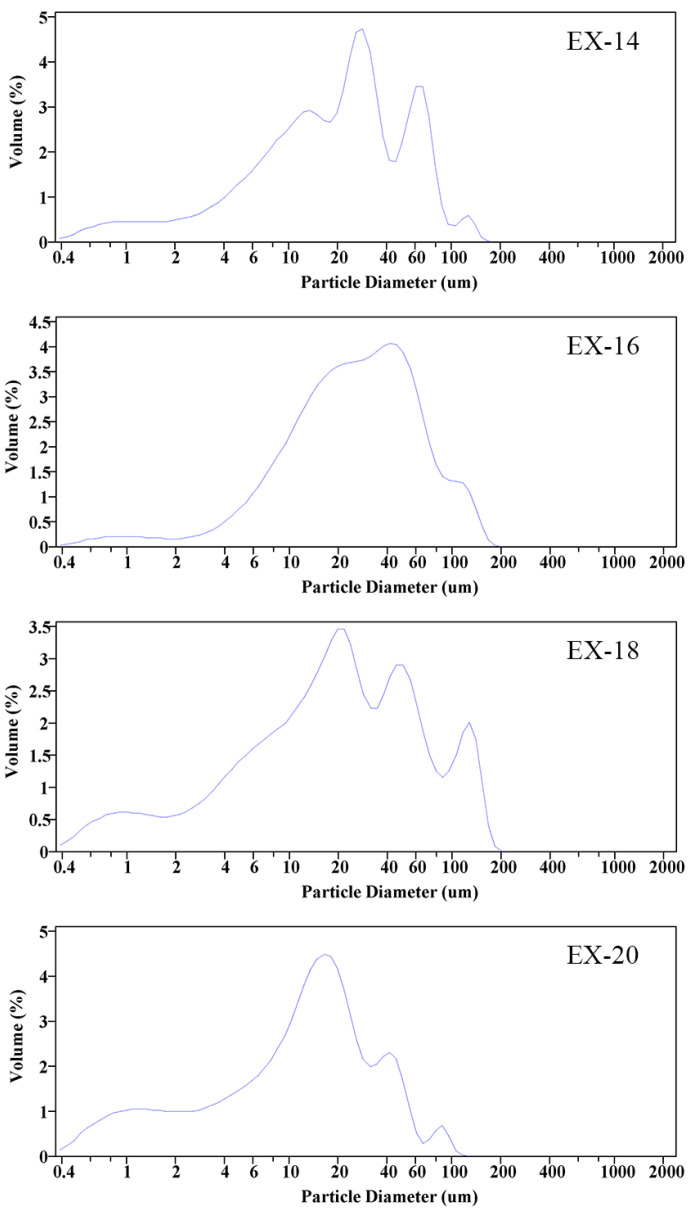
Size distributions of mAl@nAl composites under different explosion voltages.

To quantify the size distribution, mAl@nAl composites were tested by laser particle size analyzer. The size distribution curves are displayed in Figure 6. It could be learned from Figure 6 that continuous size distributions from 0.4 μm to over 100 μm were realized for all composites prepared under four voltages. It should be noted that the particle size analyzer could not tell the size of individual nAl particles from those that agglomerated together due to the limitation of the detection principle. Therefore, it was safe to say that there were particles smaller than 0.4 μm for all cases. Despite the above shortage in nano-size measurement, a particle size analyzer was still the best equipment to evaluate the continuity of the particle size distribution. It could be concluded that the size distribution of the Al particles would be significantly adjusted by changing the supplied voltage.

To compensate for the measurement shortage in the nano-scale Al particles, BET SSA and the density measurements were both used. They were both effective means to comparatively evaluate the contents of nano-sized particles of different samples based on the high specific surface area nature of nanoparticles. With the increase in nanoparticles, the SSAs of the samples would increase while their densities would decrease.

**Figure 7:**
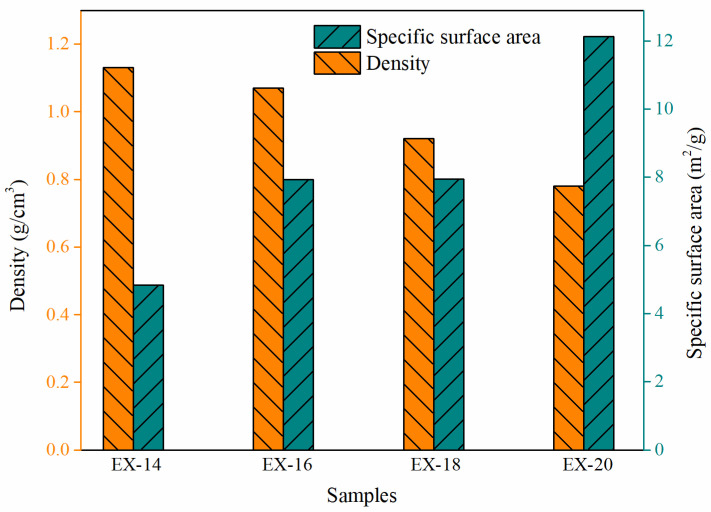
Densities and specific surface areas of mAl@nAl composites under different voltages.

The BET SSA and density results of the samples are presented in Figure 7. The BET SSAs of the samples increased from 4.8419 m^2^/g to 12.1192 m^2^/g along with the increase in voltage from 14 kV to 20 kV. Meanwhile, the densities of the samples gradually reduced from 1.13 g/cm^3^ to 0.78 g/cm^3^. It should be noted that the density of EX-14 was comparable to that of FLQT-4 (1.25 g/cm^3^), one type of aluminum product widely used in China prepared by gradation of micron and nano-sized aluminum particles. Based on the above analyses, the conclusion could be drawn that the in situ electric explosion of Al wire strategy was feasible to prepare aluminum composites with adjustable continuous size distributions, which will lead to desirable large density.

### 3.2. Reaction Performance of mAl@nAl Composites

#### 3.2.1. Combustion Efficiency of mAl@nAl Composites

The large heat release of Al particles is one of the most important characteristics that are attractive to the energetic field. The theoretical combustion heat release of Al particles is 31 kJ/g. However, due to the aluminum oxide and insufficient combustion, the actual combustion heat of Al particles is usually smaller than its theoretical heat. The combustion heat results of mAl@nAl and mAl/nAl composites are listed in Table 2. The theoretical combustion heats of the samples were calculated according to Equation (4):

*Q_s-theo_* = *Q_theo-Al_* × *w* (4)

where *Q_s-theo_* is the theoretical combustion heat of sample, kJ/g; *Q_theo-Al_* is the theoretical combustion heats of Al particles, kJ/g; and *w* is the content of active Al, %. Additionally, the combustion efficiencies of the samples were acquired using Equation (5):

*η* = *Q_s-actu_*/*Q_s-theo_* (5)

where *Q_s-actu_* is the actual combustion heat of the sample, kJ/g.

It could be learned from Table 2 that *Q_s-actu_* and *η* of physical graded samples increased with the increase in content of nAl. In the case of mAl@nAl composites, *Q_s-actu_* and *η* increased in the sequence of EX-14 to EX-18. Although *Q_s-theo_* of mAl@nAl composites was lower than that of physical graded counterparts, *η* of EX-16 was 1.27% higher than that of PG-25. The highest *Q_s-actu_* and *η* of all samples were achieved by EX-20: 10.67 and 38.67%. It was the uniformity and continuity of the size distribution that significantly affected the combustion performance of Al particles, which could not be achieved by physical gradation. Besides, the intimacy of mAl and nAl particles for mAl/nAl composites was weaker than that for mAl@nAl composites. On the contrary, the unique structure of mAl@nAl composites would enable direct heat transfer from nAl to mAl particles, which would boost the reaction of mAl particles. This result was exciting for it proved that the hierarchy structure of Al particles could increase the reaction efficiency. For the common application of Al particles, the combustion environment usually has an insufficient oxidizer supply. Therefore, it is meaningful to enhance the combustion efficiency under zero oxygen balance.

#### 3.2.2. Constant-Volume Combustion Tests under Zero Oxygen Balance

##### *P*-*t* Results

**Figure 8:**
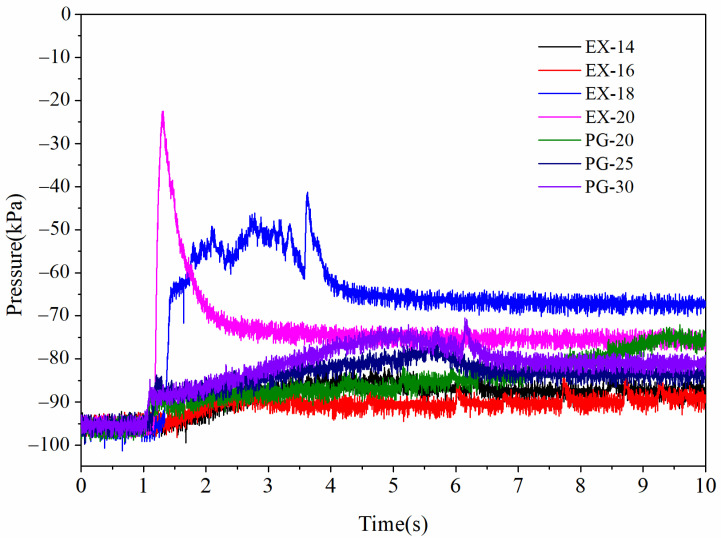
*P*-*t* profiles of samples at constant volume under zero oxygen balance.

The pressure–time (*P*-*t*) relationship of the samples was determined by measuring the generated pressure in a closed vessel during combustion as a function of time, which is an effective technology to evaluate the work capacity. The resulting pressure was significantly affected by the rate and amount of heat release from the sample reaction. The *P*-*t* results of mAl@nAl and mAl/nAl composites are shown in Figure 8.

As shown in Figure 8, there were mainly two evolving trends of pressure for the samples. The first evolving trend of pressure was slow and gentle from the beginning to the end. The pressure evolvements of physical graded samples, EX-14 and EX-16, showed the first trend. However, there was a slight difference between them. The *P*-*t* profiles of physical graded samples exhibited a slight and broad bump in the following time. The pressure bump indicated a relative concentrated release of heat. The time of occurrence of bump was related to the weight percent of nAl particles. The more the weight percent, the earlier the occurrence of the bump. By contrast, the pressurization profiles of EX-18 and EX-20 composites were completely different from the first trend. Their peak pressure and pressurization rate were both higher than the physical graded counterparts. Particularly, a prominent peak appeared in the *P*-*t* profile of EX-20 composite at the very early stage after initiation. It demonstrated that a fierce reaction occurred immediately after the ignition, which led to the release of a large amount of heat. The pressurization rate and peak pressure of EX-18 were a little slower and lower than those of EX-20 due to the lower content of nAl particles. There was no pressure peak in their *P*-*t* profiles due to the much lower content of nAl particles in the EX-14 and EX-16 composites. Based on the above analysis, it was not only the amount of nAl content but the assembly structure of nAl and mAl particles that would significantly influence the reaction rate and efficiency. The simultaneously recorded spectra (Combustion Spectra section) of those reactions would further support the conclusions.

##### Combustion Spectra

**Figure 9:**
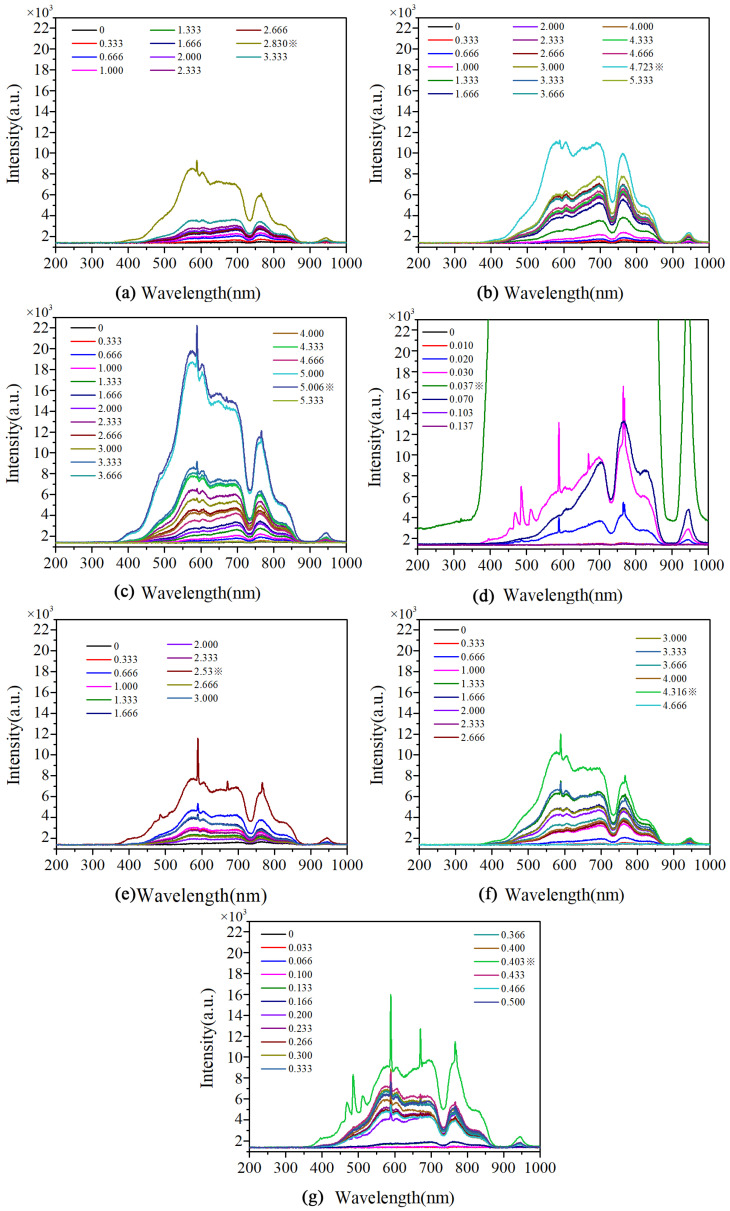
Combustion spectra of the samples: (**a**) EX-14, (**b**) EX-16, (**c**) EX-18, (**d**) EX-20, (**e**) PG-20, (**f**) PG-25, (**g**) PG-30 (the legend refers to the emission time of each spectrum in the unit of s, ※ refers to the time when strongest intensity of the spectrum acquired).

The combustion spectra of the samples are exhibited in Figure 9. To give a clear and vivid vision of the evolvement of the spectra, the spectrum of each sample from the beginning to the time when it reached to the highest intensity was listed at intervals of 0.333 s. In addition to the *P*-*t* results, the whole picture of the reaction process could be acquired. As shown in Figure 9, the highest intensity of the combustion spectra was increased in the sequence of EX-14 to EX-20, which was consistent with the *P*-*t* results. The higher the intensity of the spectra, the more sufficiently the reaction proceeded. Therefore, composite sample EX-20 showed the best thoroughness of the reaction. Besides, the time reach to the highest intensity was different for all samples. The were 2.830 s, 4.723 s, and 5.006 s for EX-14, EX-16, and EX-18, respectfully. It implied that the reaction consistency and completeness of the resulting sample would be boosted with the increase in supplied voltage. In the case of the EX-20 composite, the reaction rate was significantly improved, and it only took 0.037 s to achieve the highest intensity of the spectrum. In the following time, the intensity of the spectrum decreased immediately, which demonstrated the decline of the reaction. By comparison, the highest intensities of the spectra of PG-20 and PG-25 were comparable to those of EX-14 and EX-16, respectfully, and so was the time taken to achieve the highest intensity. Although the highest intensity of the spectrum of PG-30 was stronger than that of PG-20 and PG-25, it was still much lower than that of EX-18 and EX-20. In addition, it took 0.403 s to achieve to the highest intensity for PG-30, which was ten times longer than the time taken for EX-30. In conclusion, the addition of nAl particles could only benefit the proceeding of the reaction to a limited extent without in situ assembly of nAl and mAl. With the help of the in-situ assembly strategy, the continuous size distribution and uniform coating of nAl on the surface of mAl could be achieved, which provided a direct and intense heat and mass transfer pathway between nAl and mAl and allowed more thorough oxidation.

#### 3.2.3. Constant-Volume Combustion Tests at Constant Heat

**Figure 10:**
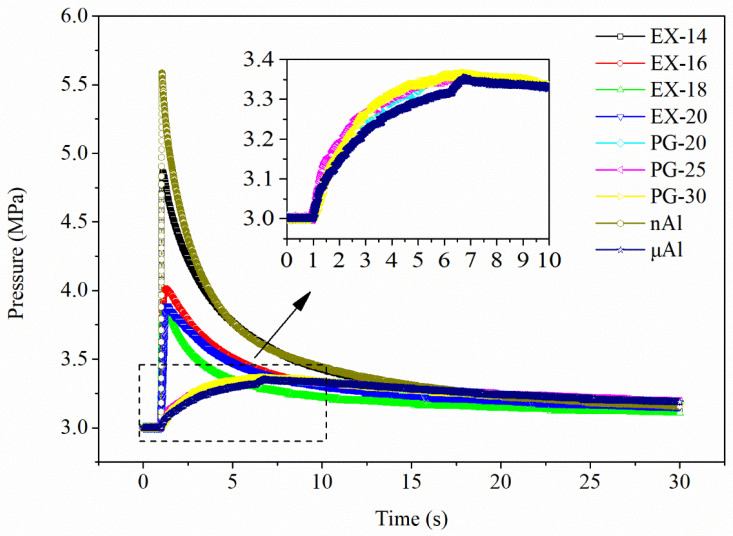
*P*-*t* curves of constant-volume combustion at constant heat.

The constant-volume combustion test at constant heat is a feasible means to conduct a comparative study on the combustion temperatures of different samples. The pressure in a constant-volume bomb is determined by the change in gas volume. If the volume of gaseous product is negligible, the change in gas volume would be only attributed to the change in temperature of the atmosphere. The oxidation product of aluminum was in a solid state, hence the relative temperature of reaction of each sample could be acquired from the *P*-*t* profile. The *P*-*t* curves of constant-volume combustion at constant heat are displayed in Figure 10. To show the difference in pressurization between in situ assembly samples and physical graded samples, the nAl particles and mAl particles (size: 5 μm) that were used in the physical graded products were also tested under the same conditions. It could be divided into two groups based on the pressurization behaviors of the samples. The pressurization behavior of one group complied with that of nAl particles, for which the pressurization time to peak pressure was negligible and the peak pressure was larger than 3.8 MPa. The pressurization behavior of the other group was in accordance with that of mAl particles, for which it took more than five seconds to reach the maximum pressure lower than 3.40 MPa. The peak pressures of mAl@nAl composites were reduced in the sequence of EX-14, EX-16, EX-20, and EX-18. The peak pressures of EX-14 and EX-18 were 4.86 MPa and 3.87 MPa, respectively, which were 1.45 times and 1.15 times that of PG-30, respectively. The consumption of oxygen was negligible compared to the large amount injected into the bomb. Therefore, it was safe to say that the temperature of the gas was proportional to the pressure based on the state equation of gas. According to the *P*-*t* results, the combustion temperatures of the mAl@nAl composites were much higher than those of the physical graded composites. This was because the reaction rate of the former was much faster than the latter, which would benefit the accumulation of heat and lead to a temperature rise. The results strongly supported the fact that the assembly structure of nAl and mAl and the continuous size distribution would greatly accelerate the oxidation process in the way that physical graded samples could not compare.

#### 3.2.4. Ignition Delay

**Figure 11:**
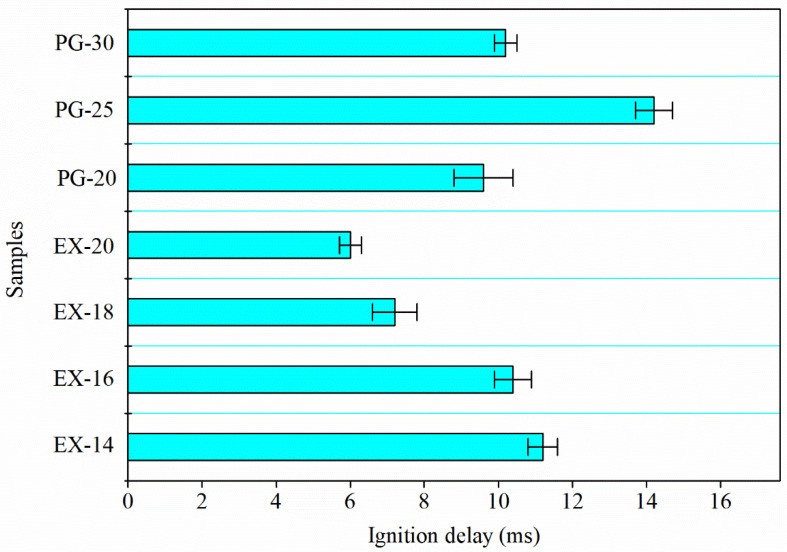
Ignition delay results of the samples under laser stimulus.

The results of ignition delay under laser stimulus are provided in Figure 11. The delay time was determined based on the interval time between laser initiation to light emitted from the sample. Therefore, the longer the delay time, the higher the energy needed to ignite the sample. It could be learned from Figure 11 that the delay time of mAl@nAl composites decreased from 11.2 ms to 6 ms in the sequence of EX-14 to EX-20. On the contrary, the ignition delay time of physically graded counterparts ranged from 9.6 ms to 14.2 ms, for which the trend of the delay time was not directly correlated with the content of nAl particles. The longest delay time belonged to the PG-25 composite. It demonstrated that random and isolated physical gradation of Al particles would not effectively improve the ignition performance. In comparison, the mAl@nAl composites possessed a hierarchy structure with continuous size distribution of Al particles from nanoscale to micro-scale. The increase in the content of nAl would directly work on the reaction performance of the composites due to the intense contact of nAl and mAl and the continuous size distribution. The two factors would both benefit and accelerate the heat and mass transfer from nAl to mAl.

## 4. Conclusions

This work addressed the paradoxical situation in the application of aluminum particles: reactivity vs. density. In situ coating of nAl on the surface of mAl particles was proposed to take advantage of the high reactivity of nAl particles while maintaining good manufacturing properties. Physical and chemical characterizations were conducted for the evaluation of mAl@nAl composites. With the in situ electric explosion of wire strategy, mAl@nAl composites with nAl adhered to the surface of mAl could be achieved. The densities of the resulting composites were comparable to those of commercial graded products. With the increase in supplied voltage, the specific surface areas of the composites gradually increased while their densities decreased. The reaction efficiency, combustion temperature, and ignition delay were quantitatively and qualitatively studied by the combustion test under zero oxygen balance, the combustion test at constant heat, and laser ignition experiments. The results revealed that continuous size distribution and the structure of mAl@nAl were the crucial factors that determine the reaction rate and efficiency. Our strategy will significantly improve the reaction performance of Al particles. We will focus on the application of mAl@nAl in explosives, pyrotechnics, and propellants in the next step.

## Figures and Tables

**Table 1:** The composition of physical graded mAl/nAl composites.

Samples	Density	Weight Percent of Each Component (%)				
	g/cm^3^	nAl	5 μm Al	10 μm Al	50 μm Al	Al Flake
PG-20	1.18	20	30	20	20	10
PG-25	1.04	25	30	20	15	10
PG-30	1.05	30	25	20	15	10

**Table 2:** Combustion heats of samples under zero oxygen balance.

Samples	PG-20	PG-25	PG-30	EX-14	EX-16	EX-18	EX-20
*Q_s-theo_* (kJ/g)	29.76	29.76	29.76	28.52	27.59	27.90	27.59
*Q_s-actu_* (kJ/g)	7.63	8.24	9.05	7.16	7.99	9.98	10.67
*η* (%)	25.64	27.69	30.41	25.10	28.96	35.77	38.67
*σ* (%)	0.33	0.17	0.30	0.42	0.33	0.36	0.36

## Data Availability

The original contributions presented in the study are included in the article, further inquiries can be directed to the corresponding author.
